# Improving Recognition and Reporting of Adverse Drug Reactions in the NICU: A Quality Improvement Project

**DOI:** 10.1097/pq9.0000000000000203

**Published:** 2019-08-30

**Authors:** Betsy Cammack, Alexandra Oschman, Tamorah Lewis

**Affiliations:** From the * Children’s Mercy Hospital, Department of Pediatrics, Division of Neonatology, Kansas City, MO; † Children’s Mercy Hospital, Department of Pharmacy, Kansas City, MO; ‡ Children’s Mercy Hospital, Division of Clinical Pharmacology, Toxicology and Therapeutic Innovation, Kansas City, MO.

## Abstract

Adverse drug reactions (ADRs) are under-recognized and under-reported in the Neonatal Intensive Care Unit (NICU) population, with up to 95% of all ADRs not reported. Compared with non-elderly adults, pediatric patients are 3 times more likely to experience an ADR, with varying rates from 0.6% to 16.8%. The Children’s Mercy NICU has an ADR rate of 0.29% (2015). This high rate presents an opportunity to increase recognition and reporting, and improve characterization of ADRs in the NICU.

**Methods:**

The primary aim of this quality improvement project was for 70% of patients who received specified medications (indomethacin, dexmedetomidine, fentanyl, lorazepam, dexamethasone, or hydrocortisone) in the first 3 months of age to be assessed daily for ADRs. We selected these medications due to the frequency of use and well-understood ADR associations. For each ADR recognized, the Naranjo score, was calculated and compared with the neonatal-specific Du score to assess the effectiveness of ADR characterization.

**Results:**

Implementation occurred on May 15, 2017. We completed 3 PDSA cycles over 1 year. The bedside monitoring tool was utilized 83% of the time. Twenty-eight potential ADRs were identified, far exceeding the number reported before implementation. The Du score appeared to better characterize ADRs compared with the Naranjo score.

**Conclusions:**

Use of a bedside monitoring tool improves ADR detection. We experienced challenges with consistently identifying patients on target drugs and getting the tool to the bedside. Application of the Du score for ADR classification in neonates appears to be more appropriate than the use of the Naranjo.

## INTRODUCTION

Neonates hospitalized in the Neonatal Intensive Care Unit (NICU) receive a multitude of medications, many of which are prescribed off-label.^1^ These medications inherently carry the potential for adverse drug reactions (ADRs), defined by the World Health Organization as “any noxious or unintended drug response at doses commonly used for prophylaxis, diagnosis or treatment of a disease or condition”.^2^ This risk is compounded due to several factors unique to neonates. All infants, and preterm infants to a greater degree have a rapidly evolving physiology with most organs and systems still undergoing a significant amount of growth and maturation. Additionally, the disease states of neonates and infants differ significantly from those of older children and adults, for whom the majority of medications have been developed and studied.

According to recent literature, ADRs are under-recognized and under-reported in the NICU population.^3,4^ When compared with non-elderly adults, pediatric patients are at 3 times higher risk to experience an ADR, with neonates and infants at a still higher risk.^3^ Incidence rates in children vary from 0.6% to 16.8%.^3,5^ Even though the risk is higher in a pediatric patient, there is limited data on the frequency, morbidity, and mortality from ADRs in pediatric patients, and even less in the NICU population. ADRs seen in children may differ from adults due to age-dependent physiological characteristics which may affect drug pharmacokinetics and pharmacodynamics. Critically ill neonates and infants are at higher risk due to the likelihood of organ dysfunction, polypharmacy, multiple classes of medications, and high medication utilization. The risk of an ADR is exponentially higher in a patient receiving ≥4 medications.^3,5^ Of neonates receiving >10 medications (significant polypharmacy), 30% of those patients experienced at least 1 ADR.^3,5^ There is a lack of understanding of the true incidence of ADRs in neonates. Based on prior published data, we suspect that they are grossly underreported.^3,5,6^

In the NICU at Children’s Mercy Hospital, ADR identification is variable. The Naranjo algorithm,^7^ a tool developed to identify and characterize ADRs in adult patients, has been used hospital-wide for the classification of potential ADRs, including in the neonatal population. This quality improvement process aims to implement the use of a novel NICU-specific ADR monitoring tool (with 70% of eligible patients showing daily use). In addition to increasing ADR detection, we will implement the 2012 Du algorithm for ADR classification.^8^ Based on prior processes, the Children’s Mercy Hospital NICU has an ADR rate of 0.29% (3 ADRs out of 1,022 admissions in 2015). When rates of admission are considered, and percentages predicted based upon the limited published data, we should be observing somewhere between 6 and 173 ADRs a year.

The primary aim was for 70% of patients who received the specified medications in the first 3 months of age to be assessed daily for ADRs within a 3-month timeframe. After completion of the first cycle and analysis of the process, we determined that the process could be improved. The second Plan-Do-Study-Act (PDSA) cycle focused on increased pharmacist involvement, and the third on implementation of an automated identification tool. The secondary aim was an improvement in our ADR reporting rate by 50% from the baseline of 3 ADRs per year. The balancing measure was the classification of ADRs using the neonatal-specific Du algorithm when compared with the Naranjo algorithm, to detect any potential discrepancies in reporting.

## METHODS

**Fig. 1.**
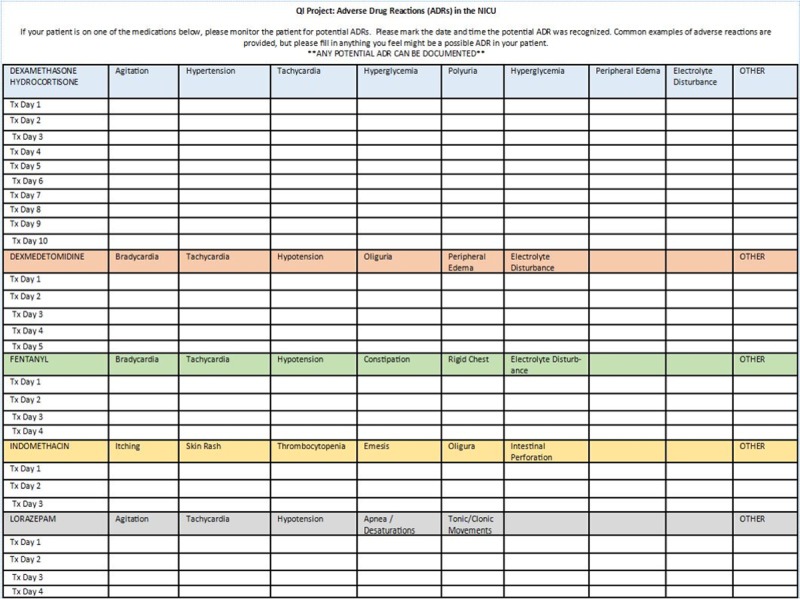
Bedside monitoring sheet.

**Fig. 2.**
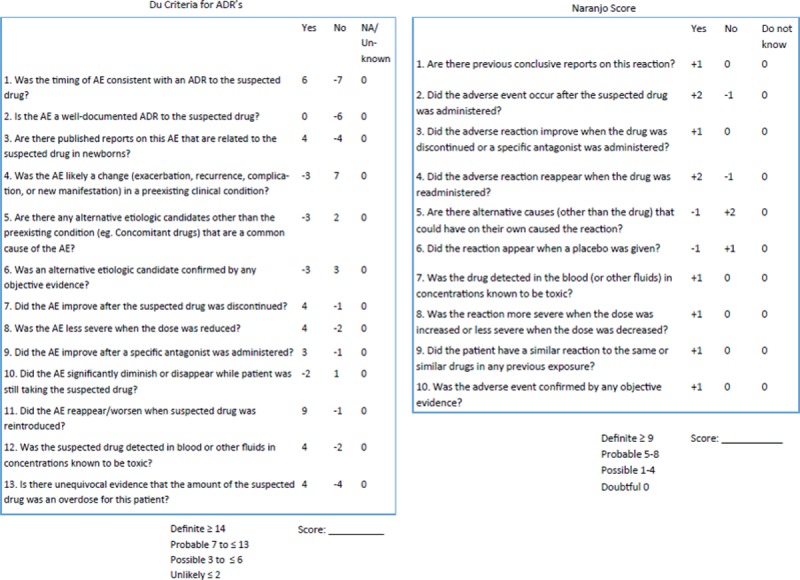
Du and Naranjo ADR scoring tools.

The Quality Improvement (QI) team consisted of a dedicated NICU pharmacist, a NICU fellow, and an attending neonatologist. The team discussed and identified barriers to detecting and discussing ADRs. Among those barriers identified were the lack of awareness of reporting systems for ADRs, time limitations during rounds, and difficulty categorizing adverse reactions as related to a particular medication. Based on these identified barriers, the team chose to implement a bedside monitoring process to test for improvement in ADR detection. Due to personnel limitations, namely a small number of people on the QI team to implement these processes throughout the large 84-bed level IV NICU, the team decided to trial the process change on a selected number of medications that were commonly utilized and had distinctive known and recognizable ADRs, which were familiar to bedside staff. The team chose 6 high-risk medications to monitor with the project: indomethacin, dexmedetomidine, fentanyl, lorazepam, dexamethasone, and hydrocortisone. We developed a bedside paper worksheet to detect and score potential ADRs (Figs. 1 and 2). For each suspected ADR, we calculated the Naranjo score and compared it with the score calculated using the Du algorithm. The institutional review board reviewed the project and determined that it was quality improvement and not human subject research. Data collection for cycle 1 occurred over 3 months, at which point results were evaluated to determine the next steps.

The day-to-day process involved the pharmacist for the team and the bedside nurse primarily. When an order was placed for one of the above-listed 6 medications, the verifying pharmacist would document some baseline demographic information and a monitoring sheet was taken to that patient’s bedside. The monitoring sheet arrives at the bedside either shortly after the drug order if it occurred in daytime hours or the next day if the drug order occurred overnight. If the nurse was not familiar with the project, the pharmacist would explain how to use the sheet to monitor for potential ADRs. When the bedside nurse identified a potential ADR, either a pre-suggested reaction or another non-suggested reaction, the nurse recorded the ADR on the bedside monitoring sheet. Nursing staff utilized their routine assessment, lab results, and vital signs to identify ADRs. They also used the Neonatal Pain, Agitation, and Sedation Score and relied on baseline patient behavior and vital sign parameters to assess reactions related to pain or sedation. The pharmacist would review the bedside sheet each morning to see if there was a potential ADR in the last 24 hours. If so, they would collect additional information, score the ADR based on the Naranjo and Du scoring systems (Fig. 2), and then discuss the potential ADR with the nurse practitioner and physician for that patient. The decision to utilize 1 pharmacist was based upon familiarity with the proposed project and proving that the concept could work before expanding the initiative. Nurse practitioners and attending neonatologists participated from a clinical decision standpoint, and it was a goal of the project for each potential ADR to be discussed by the medical team during rounds. If the team determined that the adverse event was not due to the medication, but rather from another etiology, they documented this decision on both the scoring tool and in the medical record.

Evaluation of the process took place after 3 months, and the subsequent 2 cycles were developed based on experiences and results. We anticipated that several cycles would be needed to attain our primary aim. Three cycles have been completed to date and are discussed within.

## RESULTS

The QI project launched on May 15, 2017. After 1 year and 3 PDSA cycles, we monitored a total of 124 patients for ADRs. We identified 28 patients with potential ADRs. The clinical characteristics of these patients are outlined in Table 1. The median gestation age at birth was 28 weeks, median birth weight was 820 g, and the median number of medications per patient excluding total parenteral nutrition (TPN)/lipids was 6. Patients were predominantly males and Caucasian.

**Table 1.**
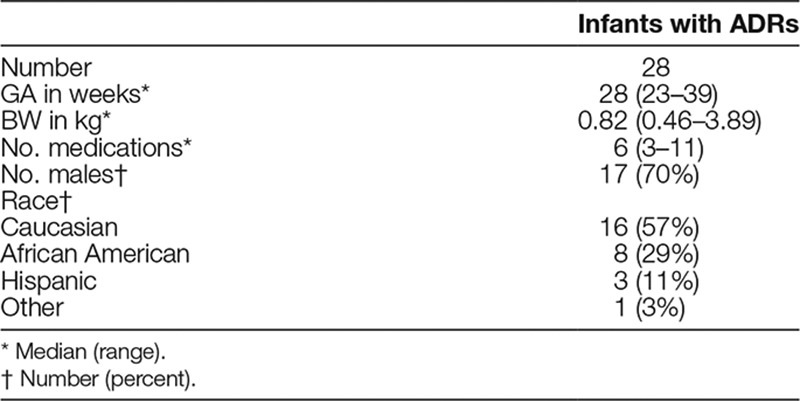
Demographics of Patients with ADRs

At the end of the first PDSA cycle, we observed that 1 pharmacist was taking on the brunt of the work with identifying patients, distributing the bedside tool, collecting the tool, inputting data, and calculating the Du and Naranjo scores. The overreliance on 1 particular NICU pharmacist led to variable compliance and analysis at the times that this particular pharmacist was unavailable. This observation highlighted the need to boost stakeholder engagement. The second cycle focused on increasing involvement of the entire NICU pharmacy staff, resulting in a shared workload. The QI team communicated with pharmacists who spent time in the NICU and devised a workflow that incorporated identification of patients and distribution of the bedside tool into the daily tasks of the pharmacists. Data collection occurred for another 3 months. There continued to be a difficulty with patient identification, resulting in several target patients who were missed and did not receive the bedside tool. The third cycle incorporated the implementation of an automated Electronic Medical Record (EMR) report that identified patients on the target medications and then sent that report to the NICU pharmacy staff twice a day.

The bedside monitoring tool was utilized 83% of the time for patients who received the noted medications, accounting for all days the patients were exposed to the selected medications. We identified 24 potential adverse reactions. Within this timeframe, there were a total of 1,013 admissions to the NICU, out of whom 124 patients were monitored. These observed results increase the overall identification rate of ADRs in our NICU from 0.29% (3/1,022) to 22% (28/124) in the population monitored. Table 2 is a summary of the adverse reactions identified according to this process. The increased identification of ADRs based on the 3 PDSA cycles represents a dramatic increase in the number of ADRs detected, suggesting a much higher rate in the overall NICU population (though not directly quantified in this project) than what is currently reported. It is important to note that application of the Du algorithm resulted in a more precise analysis of each potential adverse reaction.

**Table 2.**
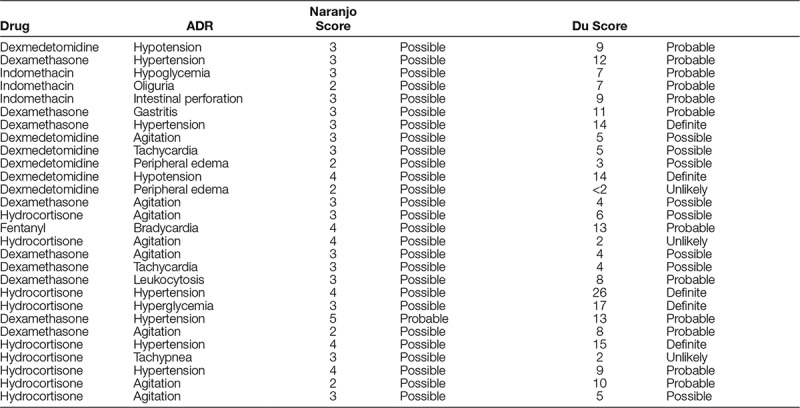
Comparison Between Naranjo Score and Du Score for Each Reported ADR

## DISCUSSION

We accomplished the primary aim of 70% utilization of the new bedside tool utilization in the first QI cycle. Analysis of the data collected suggests that ADRs are much more prevalent than previously reported and that there is potential for increased recognition of these events. During this project, we noted anecdotally that nurses and physicians were talking more frequently about ADRs, and they stopped several medications when an adverse event was recognized. Raising awareness of potential ADRs improved the rates of reporting and aided in the correct identification of each adverse event.

The rate of ADRs detected is significant. Twenty-eight ADRs out of 124 monitored patients is a rate of 23%. This ADR rate is more consistent with (and even exceeds) the published rates of ADRs in children, which ranges from 0.6% to 16.8%.^3,5^ Importantly, the adverse events ranged from expected with no intervention needed to rarer serious adverse events requiring either discontinuation of the medication or additional treatment. These data suggest that there are likely many more unidentified ADRs present in the NICU because not all patients participated in the QI project. There is a potential to capture many more adverse drug events with the implementation of this tool for all patients and all medications, thus enhancing the safety of our patients.

One of the challenges in the correct identification of ADRs in this patient population is the recognition of the ADR. ADR algorithms have been in use for decades, but do not always include confounding variables, such as disease state or concomitant use of other medications. Algorithms can also vary based on the “weight “of each variable. The majority of these algorithms, including the Naranjo, have not been assessed for validity or reliability in neonates and infants. Du and colleagues published an algorithm in 2012 by that was developed and validated for the neonatal and infant population.^8^ This new algorithm was compared with the existing Naranjo algorithm and was found to be more reliable for categorizing ADRs in a NICU population. The Du algorithm can lead to increased specificity in determining if a potential ADR is truly an ADR in the NICU population.

We found that the application of the Du algorithm to potential ADRs appears to categorize the events in a more thorough way than previous methods. Based on these data, the QI team believes that it is possible to systematically screen patients for ADRs and accurately evaluate them using the Du algorithm. Use of the neonatal specific Du algorithm may lead to increased accuracy when assessing potential ADRs, allowing the medical team to make an informed decision regarding the therapeutic utility of medications.

When designing the QI project, we considered counterbalancing measures, including increased nurse workload, increased pharmacist workload, and accuracy of ADR identification utilizing the Du score versus the Naranjo score. While the process involved did add some additional work for the bedside nurses, accounting for extra minutes spent writing down the ADR on the provided bedside sheet, we believe this is a justified addition given the previously unrecognized prevalence of ADRs and the potential harm that can come to patients should ADRs go unrecognized and unreported. The pharmacist workload proved to be significant. Each morning, pharmacists spent about 30 minutes with patients on the selected medications, taking sheets to the bedside, and entering demographic data into the monitoring spreadsheet. An additional 15–30 minutes were dedicated to bedside nursing education, with 15–30 minutes spent collecting bedside sheets and entering ADR data into the monitoring spreadsheet. The second and third PDSA cycles specifically addressed this factor, spreading the workload among all pharmacists in the NICU, and automatically generating a report to identify patients.

Challenges were present throughout this process, including the occasional loss of bedside tools, limited manpower available to implement this project in an 84-bed unit, and continued engagement from all physicians. To combat the waning engagement, we sent periodic email updates and performed in-person meetings to increase unit-wide awareness of the project and continue to stimulate engagement. As with any culture shift, it has been challenging to maintain engagement with the process among all bedside staff, NNPs, and physicians. The QI team the safety committee in our NICU will continue to highlight the importance of considering the impact of medications on our patients. Maintaining engagement through the emphasis on the importance of ADR identification and continued education for all clinical personnel is an ongoing project in the NICU. Development of an integrated ADR tracking system within the medical record is currently underway. Hopefully, this system will aid in the visibility of this initiative and engagement of the medical team. The addition of this ADR-specific EMR reporting tool will also allow us to expand to more medications and run automated reports of ADR frequency.

The ultimate goal of this QI initiative is to incorporate detection and discussion of ADRs into the daily workflow of all clinical personnel and to ingrain pharmacovigilance into the culture of the Children’s Mercy NICU. As the project currently stands, the QI team believes it is feasible to expand monitoring of ADRs to include all medications administered to all patients in the NICU, should additional resources become available. The data from 3 cycles of collection over 1 year showed that incorporating this process into a very small subset of our patients allowed for a >10-fold increase in detection of ADRs. In the future, we envision the inclusion of ADR detection into the electronic medical record, with ADRs charted as a vital sign and included in daily discussions on rounds. This addition would make ADRs an integrally monitored phenomenon of every patient.

## ACKNOWLEDGMENTS

The authors would like to thank Dr. Ronald Ariagno and the AAP Section of Neonatal Perinatal Medicine for their efforts to improve neonatal pharmacotherapy. Dr. Ariagno and Gerri Baer both contributed to the design of the QI project.
